# Identification of important modules and biomarkers in diabetic cardiomyopathy based on WGCNA and LASSO analysis

**DOI:** 10.3389/fendo.2024.1185062

**Published:** 2024-02-08

**Authors:** Min Cui, Hao Wu, Yajuan An, Yue Liu, Liping Wei, Xin Qi

**Affiliations:** ^1^ School of Medicine, Nankai University, Tianjin, China; ^2^ Department of Cardiology, Tianjin Union Medical Center, Tianjin, China; ^3^ School of Graduate Studies, Tianjin University of Traditional Chinese Medicine, Tianjin, China

**Keywords:** diabetic cardiomyopathy, WGCNA, LASSO, biomarkers, diagnosis

## Abstract

**Background:**

Diabetic cardiomyopathy (DCM) lacks specific and sensitive biomarkers, and its diagnosis remains a challenge. Therefore, there is an urgent need to develop useful biomarkers to help diagnose and evaluate the prognosis of DCM. This study aims to find specific diagnostic markers for diabetic cardiomyopathy.

**Methods:**

Two datasets (GSE106180 and GSE161827) from the GEO database were integrated to identify differentially expressed genes (DEGs) between control and type 2 diabetic cardiomyopathy. We assessed the infiltration of immune cells and used weighted coexpression network analysis (WGCNA) to construct the gene coexpression network. Then we performed a clustering analysis. Finally, a diagnostic model was built by the least absolute shrinkage and selection operator (LASSO).

**Results:**

A total of 3066 DEGs in the GSE106180 and GSE161827 datasets. There were differences in immune cell infiltration. According to gene significance (GS) > 0.2 and module membership (MM) > 0.8, 41 yellow Module genes and 1474 turquoise Module genes were selected. Hub genes were mainly related to the “proteasomal protein catabolic process”, “mitochondrial matrix” and “protein processing in endoplasmic reticulum” pathways. LASSO was used to construct a diagnostic model composed of OXCT1, CACNA2D2, BCL7B, EGLN3, GABARAP, and ACADSB and verified it in the GSE163060 and GSE175988 datasets with AUCs of 0.9333 (95% CI: 0.7801-1) and 0.96 (95% CI: 0.8861-1), respectively. H9C2 cells were verified, and the results were similar to the bioinformatics analysis.

**Conclusion:**

We constructed a diagnostic model of DCM, and OXCT1, CACNA2D2, BCL7B, EGLN3, GABARAP, and ACADSB were potential biomarkers, which may provide new insights for improving the ability of early diagnosis and treatment of diabetic cardiomyopathy.

## 1 Introduction

Diabetic cardiomyopathy (DCM) is a pathophysiological condition induced by diabetes mellitus (DM) that can lead to heart failure (HF). The initial stage of DCM is characterized by extensive hypertrophy of the heart and moderate fibrosis, leading to defects in systolic and diastolic function (1). Research has suggested that persistent hyperglycemia, insulin resistance, abnormal insulin signal transduction, impaired glucose metabolism, abnormal free fatty acid uptake, oxidative stress, increased aldosterone renin-angiotensin-aldosterone system activity, heart inflammation, and abnormal mitochondrial functions are the key determinants of the cycle and biochemical changes in disease (2). Earlier intervention in hyperglycemia can effectively maintain the normal metabolic function of the myocardium, protect myocardial tissue and preserve cardiac pump function.

The diagnosis of DCM remains a challenge, especially in asymptomatic patients. At present, the main method to detect asymptomatic DCM is echocardiography, which shows cardiac hypertrophy and dysfunction, and excludes other causes (3). However, echocardiographic features of the heart lack specificity. Biopsy is the gold standard of diagnosis, and is subject to complex procedures and high risks. In addition, specificity and sensitivity biomarkers for DCM are lacking (4). Therefore, there is an urgent need to develop useful biomarkers to help diagnose and assess the prognosis of DCM in this situation.

Weighted gene coexpression network analysis (WGCNA) is a systems biology method used to describe the correlation patterns between genes. WGCNA can be used to find clusters of highly correlated genes, aggregate such clusters with modular trait genes or in-module hub genes, and calculate module membership metrics. It can also be used to identify candidate biomarkers or therapeutic targets (5, 6). The least absolute shrinkage and selection operator (LASSO) generates a more refined model by constructing a penalty function and setting some regression coefficients to zero. Therefore, the advantage of subset shrinkage is retained, with high predictive value (7), which can improve the accuracy and predictive value of key genes identified from microarray and high-throughput data (8). In this study, WGCNA and LASSO regression were used to screen and verify the biological diagnostic markers of DCM to reduce the difficulty of screening and diagnosing DCM.

## 2 Materials and methods

### 2.1 Data source

We searched the database for myocardial sample data of mice with a high-fat diet combined with a short-term intraperitoneal injection of STZ-induced type 2 diabetes and fed a high-fat diet for more than 16 weeks, showing changes in cardiac function. As previously mentioned, a high-fat diet in mice combined with intraperitoneal injection of STZ resulted in a phenotype similar to insulin resistance and glucose intolerance used to simulate type 2 diabetes (9). After feeding a high-fat diet for more than 16 weeks, the mice developed cardiac structural dysfunction, impaired cardiac energy response, and other manifestations, and we believe that they developed myocardial damage in type 2 diabetes (10). The myocardial gene expression datasets (GSE106180, GSE161827) of the DCM group and control group were selected from the GEO database (http://www.ncbi.nlm.nih.gov/geo/) on the platform GPL19057 [Illumina NextSeq 500 (Mus musculus)].

### 2.2 Differential gene screening

We performed a homologous gene conversion with the R package “biomaRt” (11), eliminating genes that cannot translate into human genes. Log2 (x+1) processing was performed on count data and the “normalizeBetweenArrays” algorithm was used to remove the batch effect (12). Then we used the “limma” package in R to screen differentially expressed genes (DEGs). P<0.05 was set as the threshold value for DEG identification.

### 2.3 Evaluating immune cell infiltration

The genes of different types of immune cells were obtained (13). The infiltration levels of 28 immune cells were calculated using the “GSVA” R package single sample gene concentration analysis (ssGSEA) algorithm.Immune cell infiltration was compared between the DCM group and the control group. P<0.05 was considered to indicate differential infiltration of immune cells.

### 2.4 WGCNA network construction and module identification

We used WGCNA to construct gene coexpression networks and explored modules highly associated with diabetic cardiomyopathy (5). The cluster tree was constructed to test outliers. Soft threshold power was selected through network topology analysis. Then the adjacency relation was calculated, and transformed into a topology overlapping matrix (TOM). We calculated the corresponding differential degree and generated a hierarchical cluster tree of genes. Modules with similar expressions are identified and merged by dynamic tree cutting. Genes in the most important modules associated with clinical characteristics were identified for further analysis. Gene significance (GS) and module membership (MM) were used to quantitatively analyze the module genes with the highest correlation with traits. MM > 0.8 and GS> 0.2 were set as the hub gene criteria. Selected hub genes were included for further analysis.

### 2.5 Functional and pathway enrichment analysis

Gene Ontology (GO) enrichment analysis and Kyoto Encyclopedia of Genes and Genomes (KEGG) analysis were performed using the R package “clusterProfiler” (14). We used the string (V11.5, Swiss) database (string-db.org/) to build a protein-protein interaction (PPI) network and used Cytoscape Software (V3.8.2) to calculate degree scores and construct a gene association map. The minimum needed interaction score was set at 0.900 (highest confidence).

### 2.6 Screening the diagnostic markers

We searched DCM-related genes in the GeneCards Database(www.genecards.org/). Then a custom Venn diagram was drawn through the web (bioinformatics.psb.ugent.be/webtools/Venn/) to screen for the common key genes between the DCM genes, DEGs and module genes. We built LASSO models to identify key genes. The “Glmnet” package in R software was used to establish the LASSO model to identify key genes. The minimum lambda value is then used as a reference to determine the best parameter. The expression values of the selected genes were weighted using regression coefficients from LASSO analysis. The final model generated by the optimal lambda values was analyzed and the regression coefficients for each gene were calculated. After removing the gene with a coefficient of 0, we used the remaining coefficients to build a diagnostic model. The regression coefficient of hub genes was determined according to the following formula:


index=ExpGene1×Coef1+ExpGene2×Coef2+…+ExpGenen×Coefn

"Exp" refers to the expression value of a gene and "Coef" refers to the regression coefficient of the gene.” The pROC” package of R software was used to evaluate the stability and sensitivity of the LASSO model in recognizing DCM by ROC curve analysis (15).

### 2.7 Module validation and efficacy evaluation

The accuracy and validity of the model were verified by data sets of DCM mouse myocardial tissue (GSE163060) and human peripheral blood mononuclear cells (PBMCs) (GSE175988). Then we used ROC curves and AUC (Area Under Curve) to evaluate the ability to distinguish DCM from the control group with the “pROC” R package.

### 2.8 Cell culture and treatment

H9c2 cells were purchased from The Cell Bank of Type Culture Collection of The Chinese Academy of Sciences and cultured with DMEM containing 10% FBS and 1% penicillin/streptomycin at 37°C in a 5% CO2 atmosphere. We induced DCM *in vitro* by culturing H9C2 cells in 33 mmol/l glucose for 24 h.

### 2.9 Real-time quantitative PCR analysis

The total RNA of H9c2 cells was collected according to the instructions of the TRIzol reagent (Solarbio, China), and cDNA was synthesized with the reverse transcription reagent (Yeasen, China). RT-qPCR with SYBR Green detection chemistry was performed on a Roche LightCycler 96 system with the following primers (**Table 1**).

**Table 1:** Primers used for RT-qPCR amplification.

Primers	Forward	Reverse
Cacna2d2	GCCTGTGACCTTGGACTTC	CTGATCTGCTTGTGGCCTT
Bcl7b	CATCGAGAAAGTGCGGAAA	CGGAGGGAAAACCATTAGG
Egln3	TCTGTGAGCGAGATGCC	GGAAGTTGTCCAGGTAGCA
Gabarap	CCAGGAACACCATGAAGAA	GGATGCCAAGGAAGGAG
Acadsb	CCCTATGTTTCGCACCTC	CAACTTCAATGCCCATCA
Oxct1	ACATTCACCTTCCCCACA	TCCTGTTTGCCGTCTCA

### 2.10 Statistical analysis

Data analysis was performed using R4.1.0 and GraphPad Prism 8. A T-test was used to compare normally distributed data, while the Wilcoxon test was used to compare nonnormally distributed data between the control group and the DCM group. P<0.05 was considered statistically significant.

## 3 Results

### 3.1 Workflow

**Figure 1:**
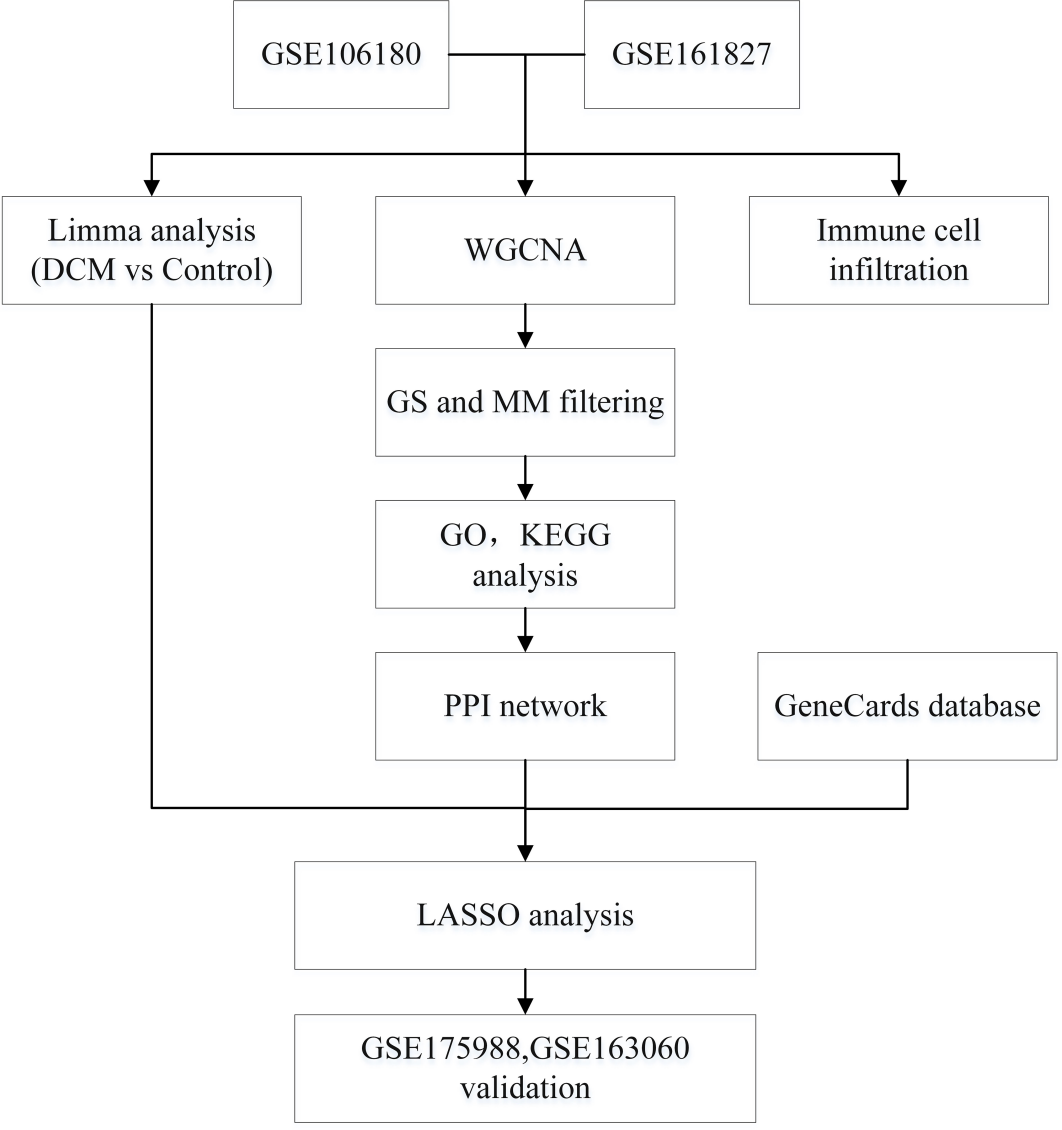
Study flowchart. Sequencing data from diabetic cardiomyopathy and controls in the GSE106180 and GSE161728 datasets were analyzed by bioinformatics to identify early potential biomarkers of diabetic cardiomyopathy. DCM, diabetic cardiomyopathy; WGCNA, weighted gene coexpression network analysis; GS, gene significance; MM, Module membership; GO, Gene Ontology; KEGG, Kyoto Encyclopedia of Genes and Genomes; PPI, Protein-protein interaction; LASSO, the least absolute shrinkage and selection operator.

The flow chart was shown in **Figure 1**. First, we compared the myocardial genes of the DCM group and the control group in the GSE106180 and GSE161827 datasets to screen out DEGs. Then we used WGCNA to construct a coexpression network and search the gene modules with the strongest correlation. Hub genes were screened by GS and MS, and we used GO analysis, KEGG analysis, and PPI network analysis to identify them. The intersection of DEGs, module key genes, and DCM was thought of as the key genes, and LASSO analysis was conducted to construct a prediction model. The ROC curve was used to test the accuracy and sensitivity of the model.

### 3.2 Identification of DEGs

**Figure 2:**
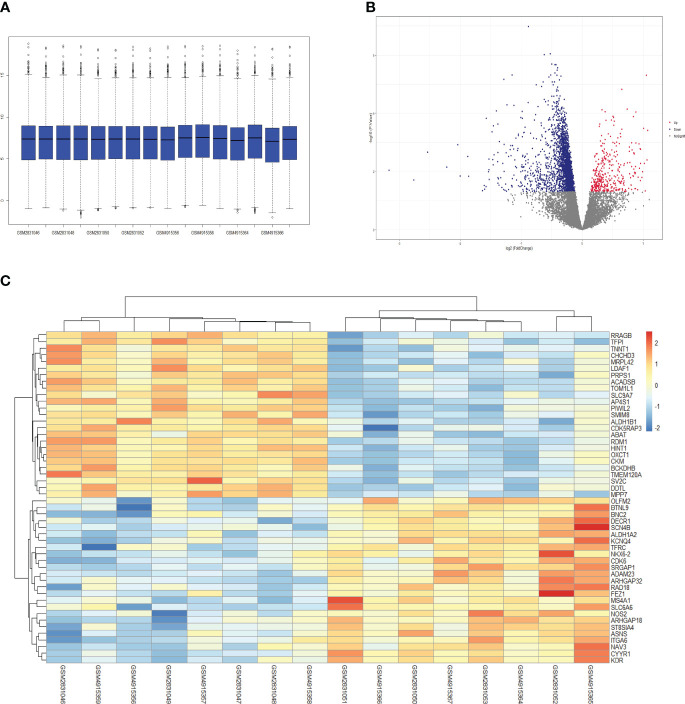
Visualization of differentially expressed genes. The red dots represent genes that are significantly upregulated and the blue dots represent significantly downregulated genes. **(A)** Box plot map **(B)** Volcano plot and **(C)** heatmap of the first 25 upregulated genes and 25 downregulated genes with minimum p values.

To compare the difference between the DCM group and the control group, we performed differential gene expression analysis. Sixteen samples were obtained from the GSE106180 and GSE161827 gene datasets, including 8 control myocardial samples and 8 diabetic myocardial samples. After adjustment, the median, upper and lower quartiles, and maximum and minimum values of genes in the 16 samples were roughly similar (**Figure 2A**). According to P< 0.05, 3066 DEGs were screened, among which 367 genes were upregulated and 2699 genes were downregulated. The first three upregulated genes with minimum p values were ALDH1A2, ADAM23, and MS4A1, while the downregulated genes with minimum p values were RRAGB, BCKDHB, and TMEM120A (**Figure 2B**; **Table 2**). The DEG heat map shows the first 25 upregulated genes and 25 downregulated genes with minimum p values (**Figure 2C**).

**Table 2:** Top 5 upregulated and downregulated genes with the most significant differences.

Genes	Regulate	logFC	P value
ALDH1A2	Up	1.050	4.92E-06
ADAM23	Up	0.648	1.51E-05
MS4A1	Up	0.736	7.23E-05
CYYR1	Up	0.323	7.58E-05
KDR	Up	0.373	8.78E-05
RRAGB	Down	-0.883	1.04E-07
BCKDHB	Down	-0.524	9.05E-07
TMEM120A	Down	-0.623	9.69E-07
OXCT1	Down	-0.501	2.04E-06
CKM	Down	-0.449	2.12E-06

### 3.3 Immune cell infiltration

**Figure 3:**
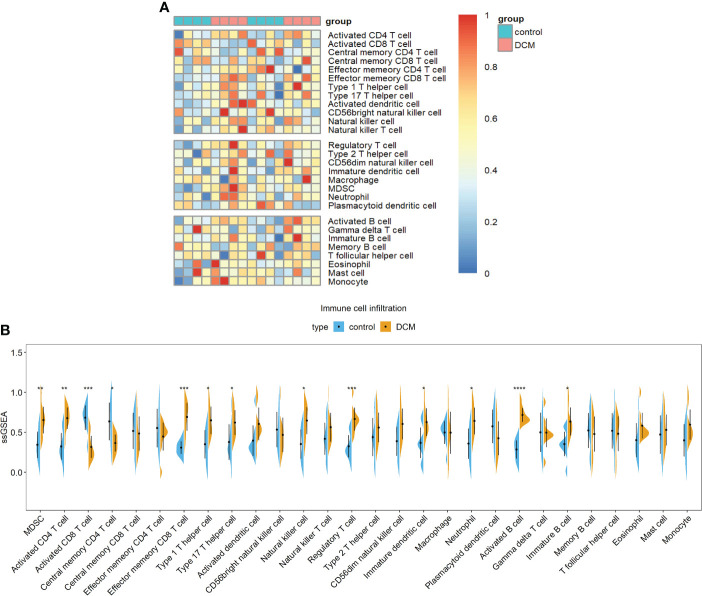
Immune cell infiltration.**(A)** heatmap **(B)** violin plot of immune cell infiltration. *p<0.05; **p<0.01; ***p<0.001. ****p<0.0001. DCM, diabetic cardiomyopathy.

Heatmaps showed differences in immune cell infiltration between the DCM group and the control group (**Figure 3A**). The violin diagram showed that MDSCs, activated CD4 T cells, effector memory CD8 T cells, type 1 T helper cell, type 17 T helper cells, natural killer cells, regulatory T cells, immature dendritic cells, neutrophils, activated B cells, and immature B cells were higher than those of the control group. In contrast, the cell infiltration of activated CD8 T cells and central memory CD4 T cells in the DCM group were less than that in the control group (**Figure 3B**).

### 3.4 DCM-associated module

**Figure 4:**
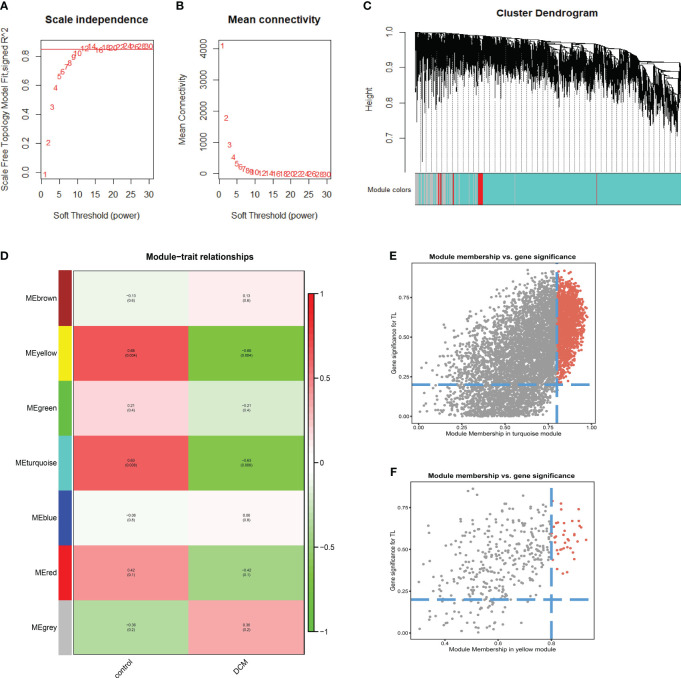
Results of weighted gene coexpression network analysis. **(A)** Scale-free fitting index (β) of soft threshold abilities **(B)** Average connectivity of soft threshold abilities **(C)** Cluster dendrogram **(D)** Module-trait relationships. Red represents a positive correlation with clinical traits, green represents a negative correlation with clinical traits **(E)** Correlation between module membership and gene significance in turquoise modules. **(F)** Correlation between module membership and gene significance in yellow modules. DCM, diabetic cardiomyopathy.

We performed WGCNA on genes in the dataset to reveal the key modules most relevant to DCM. We chose 12 as the soft thresholding power, while 0.85 was used as the correlation coefficient threshold (**Figures 4A, B, C**). After combining similar modules, we identified a total of seven modules, each shown in a different color (**Figure 4D**; **Table 3**). The yellow module with 392 genes [correlation coefficient =−0.68, P=0.004] and the turquoise module with 6471 genes [correlation coefficient =−0.63, P=0.008] are the most correlated with DCM and are the fundamental modules of DCM. According to MM> 0.8 and GS> 0.2, a total of 41 yellow module genes (**Figure 4E**) and 1474 turquoise module genes (**Figure 4F**) were included for further analysis.

**Table 3:** The characteristics of different modules.

Model	Count	Coefficient	P value
brown	619	0.13	0.633
yellow	392	0.68	0.004
green	255	0.21	0.442
turquoise	6471	0.63	0.008
blue	3788	0.06	0.826
red	176	0.42	0.107
grey	1869	0.36	0.170

### 3.5 Functional correlation analysis of genes in the key module

**Figure 5:**
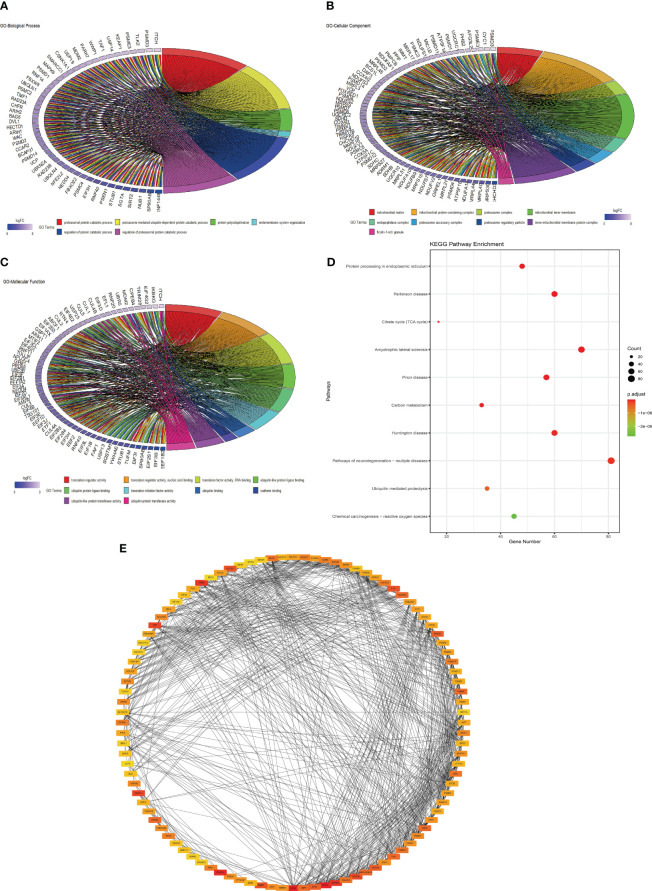
GO analysis, KEGG analysis, and PPI network of the hub genes. **(A)** Biological process; **(B)** cellular component; **(C)** molecular function;**(D)** KEGG pathway enrichment analysis; **(E)** top 100 gene network. KEGG, Kyoto Encyclopedia of Genes and Genomes.

A total of 1515 hub genes of the yellow module and turquoise module were divided into the following categories: biological process (BP) (**Figure 5A**; **Table 4**), cellular component (CC)(**Figure 5B**), and molecular function (MF) (**Figure 5C**). The GO analysis results showed that the hub genes were mainly related to “proteasomal protein catabolic process” (BP, GO:0010498, adjusted P-value = 7.79E-26), “mitochondrial matrix” (CC, GO:0005759, adjusted P value = 1.72E-21), and “translation regulator activity” (MF, GO:0045182, adjusted P value = 1.11E-16), etc. KEGG pathway enrichment analysis showed that hub genes were mainly enriched in “protein processing in endoplasmic reticulum” (hsa04141, adjusted P value = 4.47E-11), “Parkinson’s disease” (hsa05012, adjusted P value = 7.11E-10) and “citrate cycle” (hsa00020, adjusted P value = 4.27E-09) (**Figure 5D**; **Table 5**). PPI analysis showed the 100 genes with the highest score, and the top 3 genes were RPS9, FTSJ3, and PPP2CA (**Figure 5E**).

**Table 4:** Top 5 GO enriched by the yellow and turquoise module hub genes.

ONTOLOGY	ID	Description	Count	P.adjust
BP	GO:0106106	proteasomal protein catabolic process	116	7.79E-26
BP	GO:0043161	proteasome-mediated ubiquitin-dependent protein catabolic process	105	1.79E-24
BP	GO:0045333	cellular respiration	55	1.54E-15
BP	GO:0015980	energy derivation by oxidation of organic compounds	68	4.96E-15
BP	GO:0048193	Golgi vesicle transport	78	1.86E-13
CC	GO:0005759	mitochondrial matrix	106	1.72E-21
CC	GO:0098798	mitochondrial protein-containing complex	61	1.36E-12
CC	GO:0000151	ubiquitin ligase complex	63	4.82E-12
CC	GO:0005743	proteasome complex	26	2.31E-11
CC	GO:1905369	mitochondrial inner membrane	86	4.53E-11
MF	GO:0045182	translation regulator activity	49	1.11E-16
MF	GO:0090079	translation regulator activity, nucleic acid binding	41	3.58E-15
MF	GO:0008135	translation factor activity, RNA binding	35	2.07E-14
MF	GO:0044389	ubiquitin-like protein ligase binding	65	3.09E-10
MF	GO:0031625	ubiquitin protein ligase binding	62	3.54E-10

**Table 5:** Top 10 KEGG pathways enriched by the yellow and turquoise module hub genes.

ID	Description	Count	P.adjust
hsa04141	Protein processing in endoplasmic reticulum	48	4.47E-11
hsa05012	Parkinson disease	60	7.11E-10
hsa00020	Citrate cycle (TCA cycle)	17	4.27E-9
hsa05014	Amyotrophic lateral sclerosis	70	1.44E-8
hsa05020	Prion disease	57	2.51E-8
hsa01200	Carbon metabolism	33	2.79E-8
hsa05016	Huntington disease	60	8.22E-8
hsa05022	Pathways of neurodegeneration - multiple diseases	81	1.23E-7
hsa04120	Ubiquitin mediated proteolysis	35	4.69E-7
hsa05208	Chemical carcinogenesis - reactive oxygen species	45	2.75E-6

### 3.6 Screening the diagnostic markers

**Figure 6:**
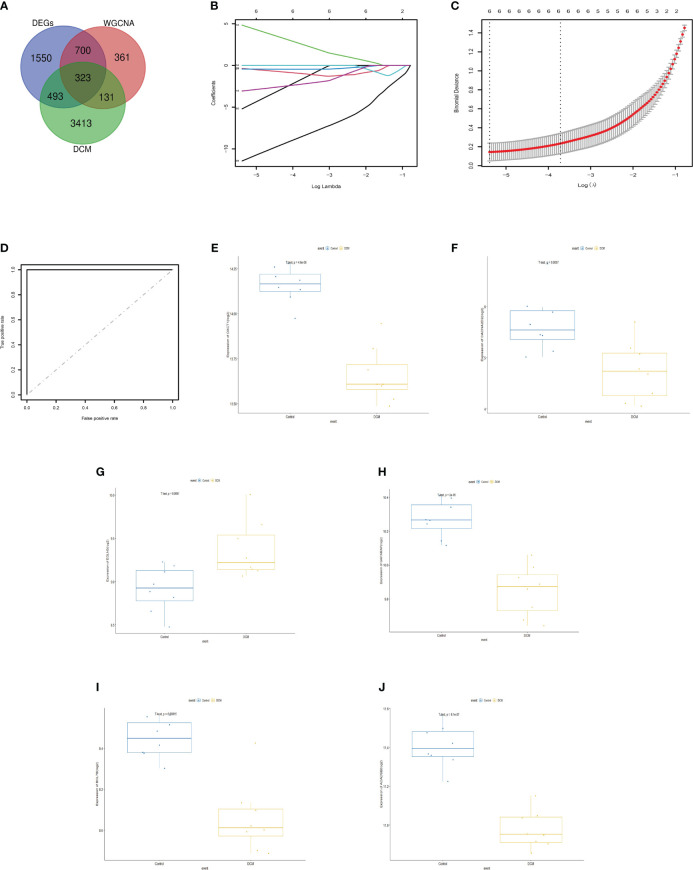
Establishment of a model for predicting DCM and its verification. **(A)** Venn diagrams; **(B)** LASSO model; **(C)**binomial deviance; **(D)** ROC curve analysis of GSE106180 and GSE161827; **(E)** Expression of OXCT1; **(F)** expression of CACNA2D2; **(G)** expression of EGLN3; **(H)** expression of GABARAP; **(I)** expression of BCL7B; **(J)** expression of ACADSB. DCM, diabetic cardiomyopathy.WGCNA, weighted gene coexpression network analysis.DEGs,differentially expressed genes.


We searched the GeneCards database for diabetic cardiomyopathy and found 4360 related genes. There were 323 genes included in all of the module hub genes and DEGs and the GeneCards database at the same time (**Figure 6A**). Then, we established a LASSO model with these selected genes based on the value of lambda (minλ= 0.00457) (**Figures 6B, C**). LASSO regression analysis was further performed, and nonzero regression coefficients were used to construct gene characteristics identified with six genes (OXCT1, CACNA2D2, BCL7B, EGLN3, GABARAP, and ACADSB). In addition, these six genes were identified based on model indices according to the following formula:

$$\begin{array}{l} \text{index}=\text{ExpOXCT}1\times (-0.9471179)\;+\text{ExpCACNA}2\text{D}2\times (-0.4471940)\;\\ \;+\text{ExpBCL}7\text{B}\times (-1.6038937)\;+\text{ExpEGLN}3\times (2.5024612)\;\\ \;+\text{ExpGABARAP}\times (-2.1876917)\;+\text{ExpACADSB}\times (-8.4851697)\;. \end{array}$$

"Exp" refers to the expression value of a gene after logarithmic conversion and coefficient refers to the non-zero coefficient calculated by the model. The DCM group tended to have higher index scores than the control group. Therefore, the calculation of the gene expression index can help diagnose DCM. In addition, we evaluated the accuracy of the LASSO model by ROC curves (**Figure 6D**), suggesting that these genes could be used as potential biomarkers for further testing of DCM. Box plots illustrate the expression trends of OXCT1 (**Figure 6E**), CACNA2D2 (**Figure 6F**), EGLN3 (**Figure 6G**), GABARAP (**Figure 6H**), BCL7B(**Figure 6I**), and ACADSB (**Figure 6J**) align with the predictions.

### 3.7 Validating the diagnostic markers in public datasets

**Figure 7:**
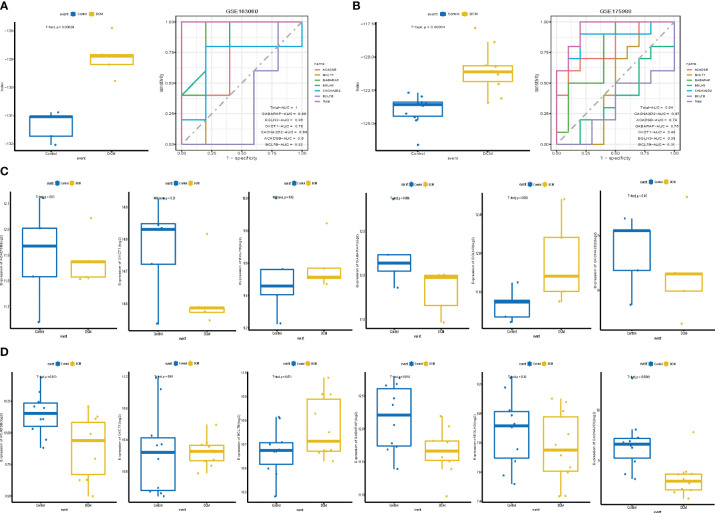
Validation of the module in Public datasets. **(A)** Box plot and roc curve of the model in GSE163060; **(B)** Box plot and roc curve of the model in GSE175988; **(C)** Box plot of the hub genes in GSE163060. **(D)** Box plot of the hub genes in GSE175988. DCM, diabetic cardiomyopathy.

A dataset containing DCM and control mouse myocardial tissue (GSE163060) was performed for human homologous gene transformation and data normalization to verify the accuracy and efficiency of the model. The results showed that the index value of the DCM group was significantly higher than that of the control group (P<0.001, **Figure 7A**). Roc curve showed that the overall AUC was 1.00 (**Table 6**). GABARAP (AUC=0.88), EGLN3 (AUC=0.86), OXCT1 (AUC=0.76), CACNA2D2 (AUC=0.68), ACADSB (AUC=0.60) had high prediction efficiency. BCL7B (AUC=0.32) was poor. The human blood PBMC cell dataset (GSE175988) was also used for verification. The index value of the DCM group was significantly higher than that of the control group (P<0.001, **Figure 7B**), and the AUC of the model was 0.94. CACNA2D2 (AUC=0.87), ACADSB (AUC=0.79), and GABARAP (AUC=0.75) had better prediction effects. OXCT1 (AUC=0.48), EGLN3 (AUC=0.38), BCL7B (AUC=0.32) was poor. There was no statistical difference in the hub genes in the mouse myocardial tissue (GSE163060) dataset, which may be related to the small sample size (**Figure 7C**). The expressions of ACADSB, GABARAP, and CACNA2D2 in the DCM group were significantly lower than those in the control group in the human blood PBMC cell dataset (GSE175988, **Figure 7D**).

**Table 6:** Validation of the hub gene in datasets GSE163060 and GSE175988.

Dateset	Genes	AUC	95%CI
GSE163060	Total	1.000	1.000-1.000
GSE163060	ACADSB	0.600	0.189-1.000
GSE163060	OXCT1	0.760	0.372-1.000
GSE163060	GABARAP	0.880	0.626-1.000
GSE163060	EGLN3	0.860	0.618-1.000
GSE163060	CACNA2D2	0.680	0.265-1.000
GSE163060	BCL7B	0.320	0.000-0.735
GSE175988	Total	0.940	0.841-1.000
GSE175988	ACADSB	0.790	0.586-0.994
GSE175988	OXCT1	0.480	0.190-0.770
GSE175988	GABARAP	0.750	0.519-0.981
GSE175988	EGLN3	0.380	0.121-0.639
GSE175988	CACNA2D2	0.870	0.697-1.000
GSE175988	BCL7B	0.310	0.066-0.554

### 3.8 Validating the diagnostic markers in H9C2 cell

**Figure 8:**
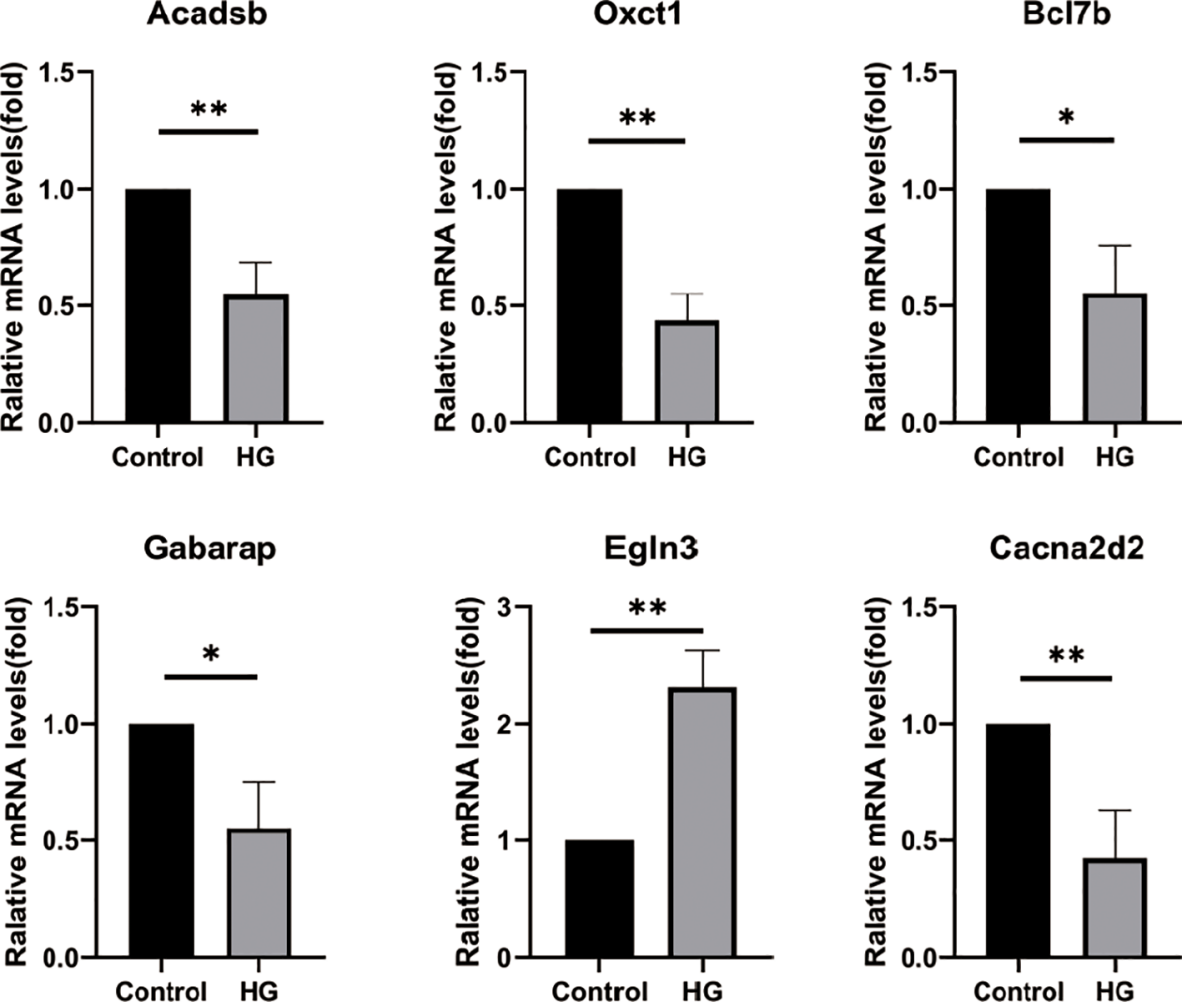
The mRNA levels of Acadsb, Oxct1, Bcl7b, Gabarap, Egln3, Cacna2d2 in H9C2 cell. HG, high glucose concentration. *P<0.05,**P<0.01.

The expression of the hub gene in H9C2 cells was verified, and the results showed that the expression of Acadsb, Oxct1, Bcl7b, Gabarap, and Cacna2d2 was decreased and the expression of Egln3 was increased in cardiomyocytes after high glucose treatment (**Figure 8**), which was similar to the previous results.

## 4 Discussion

Diabetic cardiomyopathy is a pathophysiological condition caused by diabetes that can lead to heart failure. In a series of cardiac pathologies, such as myocardial ischemia and heart failure, infiltrating immune cells are an important factor in the occurrence and development of myocardial injury and left ventricular dysfunction (16). Diabetic cardiomyopathy has attracted the attention of cardiologists and endocrinologists at home and abroad, but the diagnosis of diabetic cardiomyopathy is still a challenge, especially for asymptomatic patients. The pathogenesis, progression, and therapeutic targets of diabetic cardiomyopathy remain unknown. Therefore, it is of great significance to study the regulatory mechanisms and key targets of DCM for early prevention and treatment.

Our study selected specimens from the early stage of diabetic cardiomyopathy and found that the genes related to DCM are mainly enriched in “proteasomal protein catabolic process”, “mitochondrial matrix”, “translation regulator activity”, “protein processing in endoplasmic reticulum” and” citrate cycle” indicating that the occurrence and development of DCM are closely related to myocardial cell metabolism. Studies have shown that in patients with diabetes, the utilization rate of heart substrates is reduced, which may be due to the inhibition of fatty acid accumulation on glycolysis (17). Mitochondrial dysfunction is another cause of DCM and HF development. In general, approximately 90% of intracellular ATP production in cardiomyopathy is produced by mitochondrial oxidative phosphorylation. However, in T2DM patients, mitochondria produce ATP by oxidation of free fatty acids instead of glucose, which in turn leads to increased mitochondrial ROS production and decreased oxidative phosphorylation (17, 18). Heart failure in glycolysis and the changes in mitochondrial oxidative metabolism are due to the change in key enzymes involved in the metabolic pathway of transcription, and the NAD redox state (NAD and nicotinamide adenine dinucleotide level) and the change in metabolite signaling. Epigenetic changes after these signals will help translation control coding energy metabolism enzyme-encoding gene expression (19). Therefore, improving the energy metabolism pathway of diabetic cardiomyopathy is a new treatment direction.

After WGCNA and LASSO analysis, we found diagnostic and predictive biomarkers of type 2 DCM, including OXCT1, CACNA2D2, BCL7B, EGLN3, GABARAP, and ACADSB. However, the relationship and mechanism between these genes and DCM have not been fully confirmed. OXCT1 is a member of the OXCT1 gene family that encodes 3-oxyacid coenzyme A-transferase. This coding protein is a homologous dimer mitochondrial matrix enzyme that plays a core role in the catabolism of ketone bodies by catalyzing the reversible transfer of coenzyme A from succinyl-CoA to acatalectic acid (20). OXCT1 plays an important role in heart failure and diabetes (21, 22). Studies have shown that hyperglycemia reduces the expression of Bdh1 and Oxct1 in the hearts of mice. BDH1 and OXCT1 are also inhibited in the failing heart of diabetic patients but not in nondiabetic patients (23), which is similar to our findings. Chronic elevation of circulating ketone inhibits the development of inflammatory heart failure (22). Therefore, OXCT1 may be an important diagnostic and therapeutic target for diabetic cardiomyopathy. CACNA2D2 encodes a subunit of the voltage-dependent calcium channel complex, plays an important role in complex assembly and membrane localization, and regulates calcium current and channel dynamics. Studies have shown that high glucose induces mitochondrial fission through the Orai1 calcium channel and participates in diabetic cardiomyopathy hypertrophy (24). Calcium channel signal transduction is linked to mitochondrial function. Calcium overload leads to decreased mitochondrial membrane potential and affects cell function (25). BCL7B has some roles in maintaining nuclear structure and is involved in the regulation of multiple pathways, including Wnt and apoptosis (26). EGLN3 participates in the activation of cysteine-type endopeptidase activity during apoptosis. A study by Xia et al. showed that inhibition of EGLN3 ameliorates cardiac dysfunction in diabetic cardiomyopathy (27). The presence of GABARAP is very important for Golgi body morphology. In addition, studies have shown that it is involved in autophagy and may play an important role in diabetes (28, 29). ACADSB, a member of the acyl-CoA dehydrogenase family, catalyzes the dehydrogenation of acyl-CoA derivatives in fatty acid or branched amino acid metabolism. It plays an important role in the energy homeostasis of pathophysiological processes (30).

The hub genes in multiple datasets and cardiomyocytes were verified, and the results all showed that the hub genes had good diagnostic efficacy. Notably, similar results were obtained in the human blood PBMC dataset. So, the relevant genes could be detected in the blood PBMC, which significantly reduces the difficulty of detection compared to traditional biopsies. DCM lacks specific diagnostic criteria. As an invasive examination, myocardial biopsy has disadvantages such as high risk and complicated procedures. In addition, when DCM develops to a certain stage the heart enlarges significantly and the heart function declines, myocardial biopsy is not suitable (31). In this case, the detection of blood PBMC has the advantages of convenience, low risk, and small damage. ROC curves showed that GABARAP, EGLN3, OXCT1, CACNA2D2, and ACADSB had high detection accuracy in myocardial tissue. CACNA2D2, ACADSB, and GABARAP are more accurate in human blood PBMCs. When the economic budget is insufficient to carry out multi-gene sequencing, genes with higher diagnostic efficiency can be preferentially selected for detection by RT-qPCR. At present, the diagnosis of DCM is mainly based on myocardial biopsy, combined with a variety of detection methods. RT-qPCR is relatively cheap and therefore more suitable for screening in the population.

In addition, our study found changes in immune infiltration in cardiomyocytes in diabetic cardiomyopathy. A large number of studies have shown that white blood cells and their subgroups, such as neutrophils, monocytes, and lymphocytes, are involved in inflammation and play an important role in the occurrence and progression of cardiovascular diseases (32). Th and Treg subgroups are involved in inflammation, insulin resistance, and vascular changes in diabetes (33). Compared with healthy controls, neutrophils promote the secretion of cytokines and growth factors in diabetic patients, which helps neutrophils further migrate to inflammatory sites and promote the production of reactive oxygen species and apoptosis (29). In T2DM, Treg cells can inhibit Th1, Th2, and Th17 responses through various pathways, such as inhibiting cytokine secretion, regulating the microenvironment, and changing the expression of surface receptors to improve insulin resistance (34). B lymphocytes are antigen-presenting cells and autoantibody secretions. B-cell-deficient mice showed less inflammation and improved glucose tolerance (35). Further study of these mechanisms may contribute to the search and development of new therapeutic targets.

The strength of this study lies in the development of a diagnostic and predictive model for diabetic cardiomyopathy. The results were verified in datasets and cardiomyocytes. The model was also found to have a good predictive effect in the PBMC datasets, which means that the diagnosis of DCM can be made by blood, making detection more convenient.

Our study also has some limitations. Since it is difficult to obtain cardiac tissue samples from diabetic cardiomyopathy patients, this study only analyzed the heart tissue of diabetic cardiomyopathy animals. Second, the public datasets we analyzed did not have the results of cardiac function and pathological tests. Therefore, we cannot compare the results of this study with other models. In addition, we did not verify the diagnostic efficiency of the genes involved in PBMC in the population. Therefore, large-scale clinical studies are needed to further validate the model in the future.

## 5 Conclusion

We found diagnostic and predictive biomarkers of type 2 DCM consisting of OXCT1, CACNA2D2, BCL7B, EGLN3, GABARAP, and ACADSB, which may reduce the difficulty of early prediction of diabetic cardiomyopathy, and lay a foundation for prospective research. Our analyses provide a reference for the detection of promising biomarkers and therapeutic targets and for improving the ability to diagnose and treat patients with DCM.

## Data availability statement

The original contributions presented in the study are included in the article/supplementary material. Further inquiries can be directed to the corresponding author.

## Ethics statement

Ethical approval was not required for the studies on animals in accordance with the local legislation and institutional requirements because only commercially available established cell lines were used.

## Author contributions

Conceptualization, MC, and HW. Methodology, YA. Validation, HW, YA, and YL. Data curation, HW, and YL. Writing—original draft preparation. MC. Writing—review and editing, LW and XQ. Visualization, MC. Supervision, LW. Project administration, XQ. All authors contributed to the article and approved the submitted version.
